# Biodegradable Polyesters: Approaches to Increase Degradation Rates for Biomedical Applications

**DOI:** 10.1021/acsmacrolett.5c00417

**Published:** 2025-08-10

**Authors:** Courteney T. Roberts, Melissa A. Grunlan

**Affiliations:** † Department of Biomedical Engineering 14736 Texas A&M University, College Station, Texas 77843-3003, United States; ‡ Department of Biomedical Engineering, Department of Materials Science & Engineering, Department of Chemistry Texas A&M University, College Station, Texas 77843-3003, United States

## Abstract

The rate of biodegradation of polyesters is essential to their utility in biomedical applications but is frequently undesirably slow, prompting significant interest in overcoming this limitation. Herein, we highlight passive, enzyme-mediated, and load-mediated mechanisms of the hydrolytic degradation of polyesters. Exemplified by recent reports, strategies to impart accelerated rates of degradation are discussed, including synthetic routes, 3D systems, and processing methods. Approaches to assess polyester degradation *in vitro* and *in vivo* are summarized, underscoring the need for careful consideration of testing parameters and the challenges arising from testing variability employed within the reported literature. Recent reports also highlight faster-degrading polyester systems for targeted biomedical applications, including regenerative engineering, drug delivery, women’s health, and other medical devices. Overall, polyesters with accelerated rates of degradation will afford tremendous opportunities in bioresorbable devices and therapeutics.

## Graphical abstract

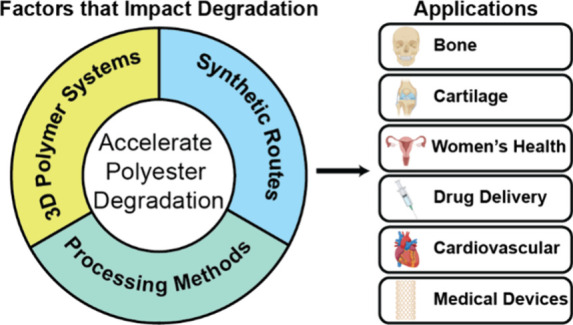

## 1 Introduction

Biodegradable polyesters,^1–3^ with resorption occurring after performing a given function, are utilized in numerous biomedical applications (e.g., medical devices, drug delivery, and tissue regeneration).^4,5^ Several biodegradable polyesters have been of particular focus (Table 1) and may be broadly classified as natural or synthetic depending on their origin.^6–8^ The degradation behavior stemming from chain scission of ester linkages is associated with a particular metabolic path to eliminate the resulting oligomeric and small-molecule byproducts. Degradation rates vary widely among these polyesters and are integral to the utility and success in a given application.^6,9,10^ Ideally, degradation rates should parallel physiological processes such as tissue healing or a targeted release profile of therapeutics. Premature resorption is undesirable and would, for instance, contribute to compromised tissue healing processes^11–13^ or toxicity effects of therapeutics. However, conventional biodegradable polyesters typically exhibit unfavorably slow rates of degradation, contributing to diminished results. For instance, polyesters frequently utilized as bioresorbable bone fixation plates are resorbed much slower versus bone healing.^14^ Likewise, the slow degradation rate of polyester bone tissue scaffolds inhibits neotissue ingrowth and integration.^11,15,16^ Thus, tuning and particularly accelerating the degradation rates of polyesters are paramount. Herein, we describe the biodegradation behavior of polyesters, including mechanisms, characterization, and approaches to accelerate rates of degradation and also highlight targeted biomedical applications.

**Table 1:**
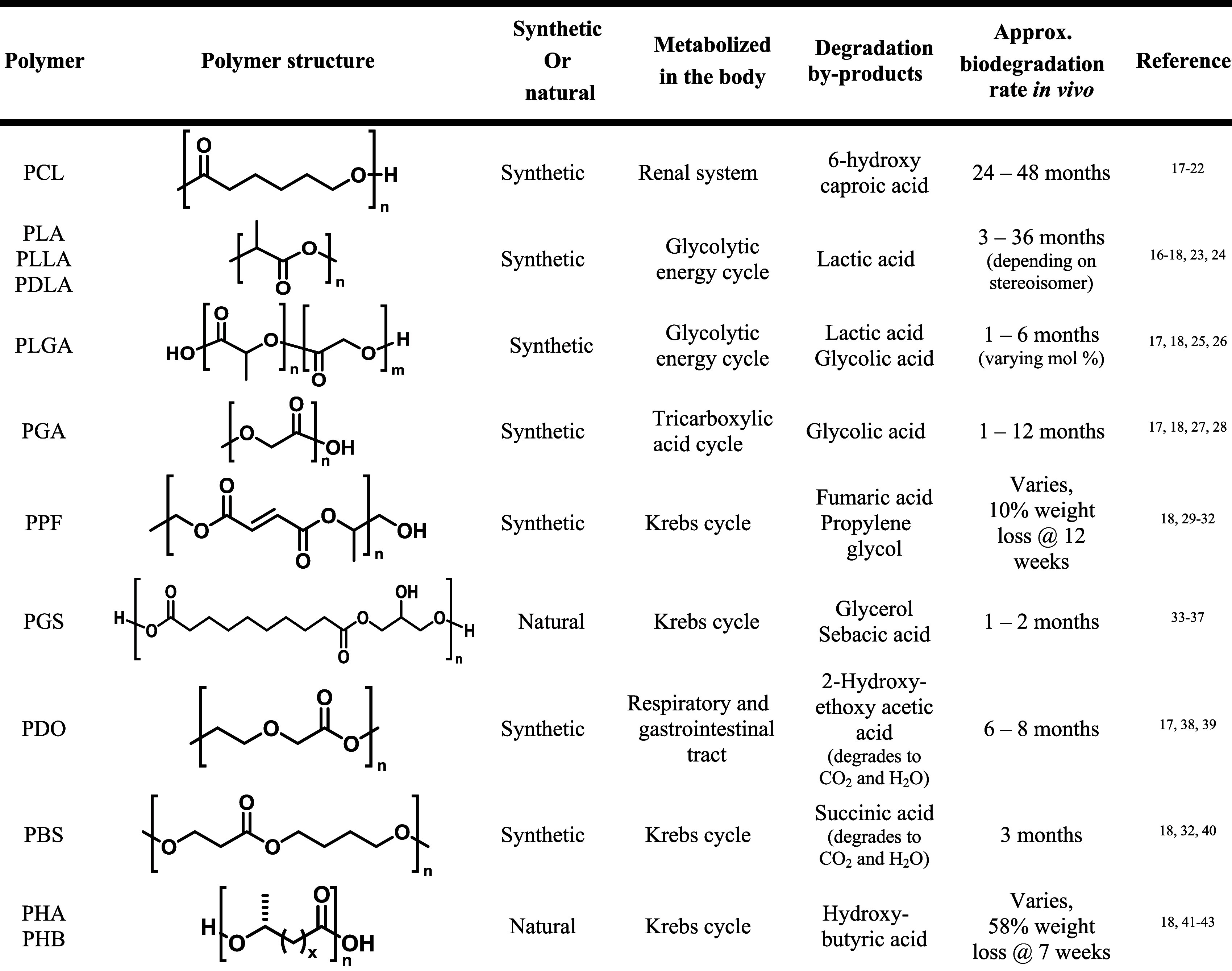
Biodegradable Polyesters^17–43^ Commonly Used in Biomedical Applications^a^

a **PCL:** Polycaprolactone; **PLA:** Poly­(lactic Acid); **PLLA:** Poly­(L-lactic Acid); **PDLA:** Poly­(D,L-lactic Acid); **PLGA:** Poly­(lactic-co-glycolic acid); **PGA:** Polyglycolide; **PPF:** Poly­(propylene fumarate); **PDO:** Polydioxanone; **PBS:** Poly­(butylene succinate); **PHA:** Polyhydroxyalkanoate; PHB: Polyhydroxybutyrate

## 2 Hydrolytic Degradation of Polyesters

The *in vivo* resorption of polyesters occurs via the cleavage of ester bonds, leading to reduced molecular weight and subsequent mass loss. Hydrolytic degradation is the primary mechanism among biodegradable polyesters, and this may be passive or catalyzed enzymatically and/or physically (Figure 1).^44–47^ While these are distinct mechanisms of hydrolytic degradation, they often occur simultaneously in the complex *in vivo* environment. (Note: oxidative degradation is generally minimal due to the high amount of radical species needed to degrade polyesters and is more prevalent in polymers with double bonds, ether linkages, and phenol moieties.^44,47^ )

**Figure 1:**
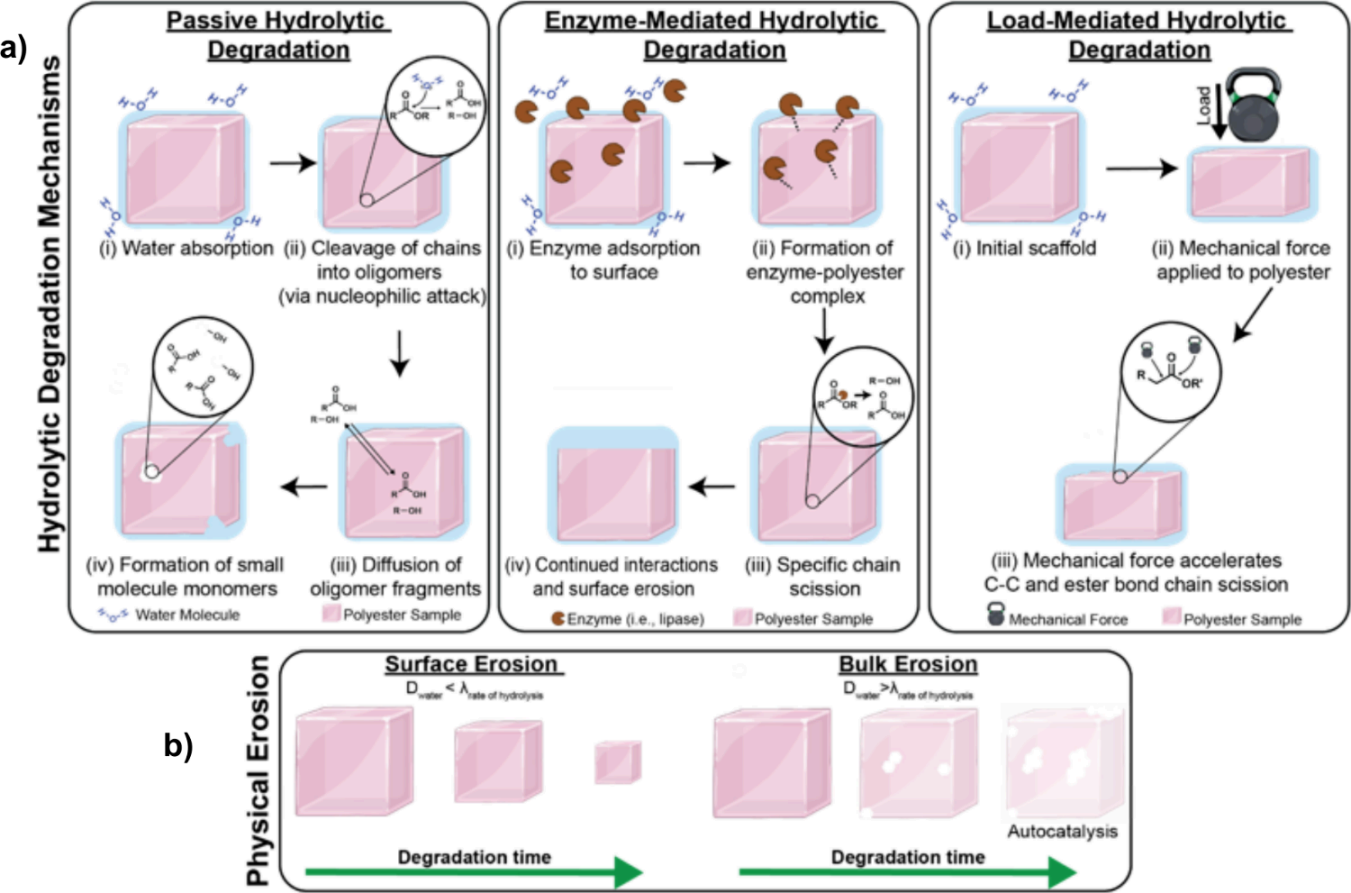
(a) Schematic of polyester hydrolytic degradation mechanisms that occur *in vivo*: passive, enzyme-mediated, and load-mediated. (b) Schematic of surface and bulk erosion processes that occur with hydrolytic degradation.

### 2.1 Passive Hydrolytic Degradation

Constituting ∼60–70% of the human body, water is present throughout the body.^48^ Thus, water-mediated hydrolysis of ester linkages is a primary mechanism of biodegradation within polyesters,^3^ as well as for polymer types comprised of other hydrolytically labile bonds (e.g., amide, anhydride, ether, ortho-ester, thio-ester, urea, and urethane/carbonate). Ester bonds of sufficiently hydrophilic polyester backbones are cleaved to form alcohols and acidic byproducts (Table 1).^3,10^ While these acidic byproducts may limit cellular growth and differentiation, and also lead to local inflammation of tissues,^4,23,49,50^ they continue to be broadly employed in biomedical applications.

Passive hydrolytic biodegradation of polyesters occurs through a series of steps: (1) water absorption; (2) cleavage of ester bonds in backbone to form oligomer fragments, and formation of smaller, oligomeric fragments; (3) diffusion of these fragments from the bulk or surface; and (4) formation of monomeric and small molecule byproducts via additional ester bond hydrolysis (Figure 1a).^51,52^ During the first phase of hydrolytic degradation, there is an initial lag (i.e., incubation phase) where absorbed water produces no significant degradation.^53^ The second phase demonstrates a gradual reduction in molecular weight without mass loss, and the third and final phase marks the beginning of mass loss and the diffusional loss of oligomers or monomers.^54^ Overall, the extent of the water interaction plays an integral role in the rate of passive hydrolytic degradation of polyesters. This is dictated by numerous factors, including the molecular structure of the polyester. For instance, ester bond location impacts the susceptibility to hydrolytic degradation with exochain (i.e., end chain) esters demonstrating higher cleavage rates than endochain (i.e., middle chain).^3,10,54^ Other factors such as hydrophilicity and crystallinity also have pronounced effects on water interaction, and hence ester bond hydrolysis.^55,56^

### 2.2 Enzyme-Mediated Hydrolytic Degradation

Degradation of polyesters can also occur via enzyme-mediated hydrolysis.^57,58^ A wide variety of hydrolase enzymes (e.g., esterase, lipase, protease, and dehydrogenase) participate in the enzymatic degradation of polyesters.^44,59,60^ Present in the liver, skin, and plasma, esterase preferentially hydrolytically cleaves ester bonds, resulting in the formation of a carboxylic acid and alcohol byproduct.^59,61,62^ Lipase, present in the pancreas, mouth, and digestive tract, hydrolyze ester bonds on water-insoluble chains (e.g., long chain).^59,63,64^ Proteases, known to hydrolytically cleave peptide bonds and to participate in various physiological processes (e.g., nutrient digestion and cell differentiation), have also been shown to cleave ester bonds, particularly L-stereoisomers of poly­(lactic acid) (PLA) due to their similarity to amino acid l-alanine.^65–67^ Enzyme-mediated hydrolytic degradation also occurs in a sequence of steps: (1) water absorption and enzyme adsorption, (2) formation of an enzyme–polyester complex, (3) chain scission of esters bonds, and (4) continued interactions between the enzyme–polyester complex (Figure 1a).^44,68^

*In vitro* testing of polyester degradation via enzyme-mediated hydrolysis is limited by an inability to recapitulate physiological conditions that include numerous enzymes of fluctuating concentrations.^46,69^ Still, several reports have sought to understand the potency of different enzymes to mediate the hydrolysis of various polyesters. Shi et al. analyzed poly­(butyl succinate) (PBS) and PLA blends in proteinase K (i.e., protease) solutions and reported that the PLA component was degraded by this enzyme while PBS was not.^70^ Seok et al. analyzed PLLA degradation in proteinase K as well as in β1,3-glucanase solutions and observed degradation of poly­(L-lactic acid) (PLLA) only with proteinase K.^71^ Keridou et al. utilized different lipases (i.e., *Pseudomonas cepacia* and *Rhizopus oryzae*) in poly­(hydroxybutyrate) (PHB) degradation and observed more weight loss in enzymatic versus nonenzymatic hydrolytic degradation.^72^ Similarly, Zhuikov et al. reported faster degradation in PLA and PLA/PHB blends in enzymatic conditions versus in nonenzymatic conditions.^73^ Rosato et al. employed several lipases, a proteinase, and cutinase to observe *in vitro* degradation differences among various polyesters.^59^ For instance, polycaprolactone (PCL) degraded the fastest in lipase from *Candida sp*. and more slowly in lipase from *Rhizopus oryzae*. Overall, bacterial (e.g., *Pseudomonas* and *Lactobacillus*) and fungal (*Candida*, *Aspergillus*, and *Rhizopus*) lipases were among the most potent in the degradation to polyesters.

### 2.3 Load-Mediated Hydrolytic Degradation

Load-mediated hydrolysis (i.e., mechano-hydrolysis), stemming from forces experienced from the surrounding physiological environment, can also contribute to the overall hydrolytic degradation of polyesters (Figure 1a).^46,74–76^ Although mechanical loads cannot initiate hydrolytic degradation of polyesters, they often accelerate the process.^75,77^ Numerous *in vitro* studies have observed this phenomenon upon subjecting polyesters to a variety of mechanical loadings in an aqueous environment. For instance, Wang et al. demonstrated that ultrasonication led to cleaving of polymer chains into smaller fragments via homolytic carbon–carbon chain scission and accelerated PCL hydrolytic degradation.^78^ Jin et al. observed accelerated hydrolytic degradation of poly­(glycerol-dodecanoate) (PGD) when subjected to tensile loading.^79^ Wu et al. subjected PGS specimens to varying tensile loads, with greater mass loss coinciding to higher loading.^80^ Díaz et al. reported a progressive reduction of compressive modulus and yield stress of PLLA/nanohydroxyapatite (HA) composite scaffolds and films.^81^ In contrast, if mechanical loading is accompanied by chain alignment (e.g., crystallization), water uptake, and hydrolysis may be reduced. For instance, Wang et al. investigated highly crystalline PLA monofilaments and showed that amorphous tie chains hydrolyzed into shorter chains and became more crystalline during degradation and that such chains were more resistant to hydrolysis.^82^ Zhao et al. observed that polydioxanone (PDO)/PCL stents subjected to static compressive loading degraded faster versus when not loaded, while dynamic loading reduced the degradation rate owing to a reduction of polymer chain viscous flow.^83^ The *in vitro* analysis of load-mediated hydrolysis is challenging given the complex and varied nature of physiological loading; however, recent research efforts have begun to explore this phenomenon.^75,84–86^

### 2.4 Physical Erosion

The hydrolytic degradation of polyesters and other polymers gives rise to physical erosion and may be categorized as either surface erosion or bulk erosion based on the characteristics of mass loss (Figure 1b). The type of erosion observed is largely dependent on the relationship between the rates of hydrolysis (*λ*_rate of hydrolysis_) and water diffusion coefficient (*D*_water_).^87^ Surface erosion (*λ*_rate of hydrolysis_ > *D*_water_) occurs at the specimen surface via an erosion front while bulk erosion (*λ*_rate of hydrolysis_ < *D*_water_) occurs throughout the specimen. Thus, while surface erosion produces a progressive decrease in dimensions, bulk erosion frequently leads to pitting and cracking, which can dramatically reduce mechanical integrity, even with low mass loss. Degradable aliphatic polyesters are known to exhibit bulk erosion wherein the entrapment of acidic byproducts accelerates the rate of hydrolytic bond cleavage (i.e., autocatalysis).^87,88^ Apart from polyesters with appreciable hydrophobicity,^89^ conventional polyesters may exhibit surface erosion in certain scenarios that afford ester hydrolysis that is more rapid than water diffusion. These include high specimen surface area^90^ and overall size,^3,91,92^ as well as exposure to highly basic media^93^ or other conditions that afford mobilization of hydrophilic moieties to the exposed surface.^94^ In the case of enzyme-mediated hydrolytic degradation, the limited diffusion of enzymes into the specimen bulk can also lead to surface erosion.^16,44,95^ However, during bulk erosion, enzymes may also penetrate the bulk via eroded pathways. Mechanical loading can also lead to surface erosion. For instance, Jin et al. observed that mechanical loading caused inconsistent degradation patterns and accelerated surface erosion of the PGD samples in an aqueous environment.^79^ Overall, the observed erosion that accompanies hydrolytic conditions is dependent on numerous factors, and care should be taken in understanding the impact of testing parameters.

## 3 Tuning Polyester Degradation Rates

To enhance their utility in biomedical applications, various approaches have been employed to accelerate the rate of polyester hydrolytic degradation, including synthetic routes (i.e., polymer structure modifications), processing methods, and 3D polymer systems (Figure 2). Recent reports of such strategies are exemplified herein.

**Figure 2:**
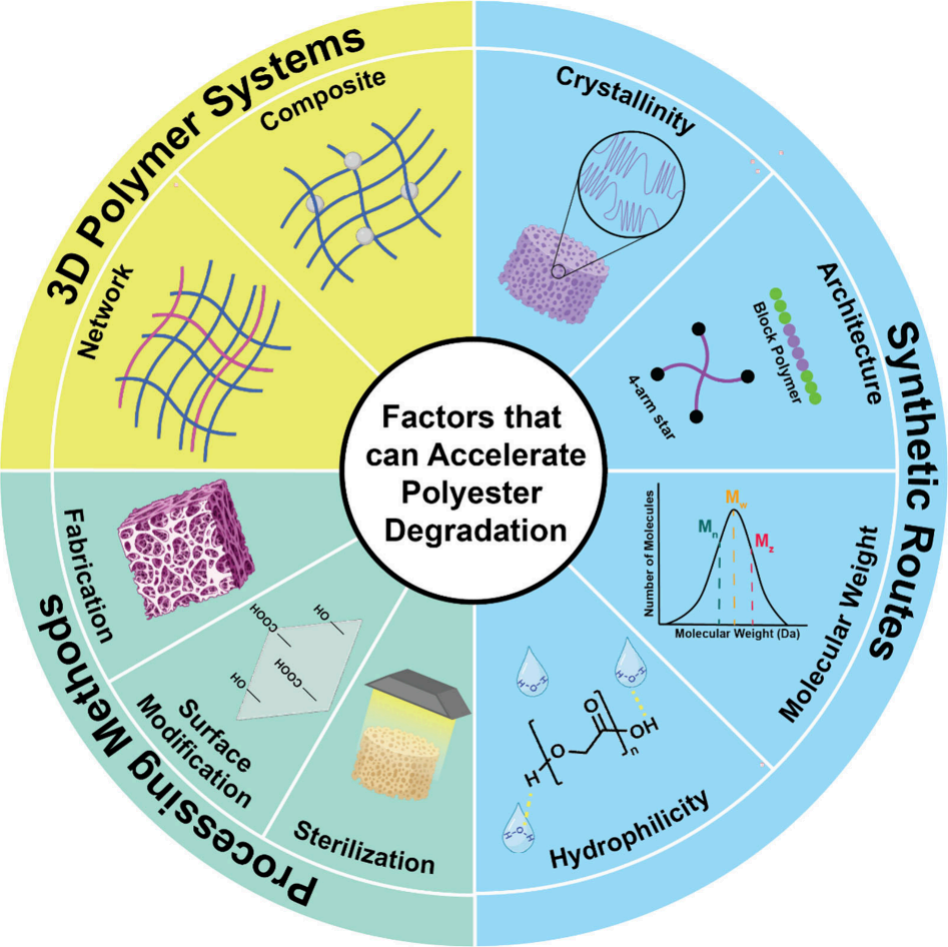
Various approaches alter the rate of polyester degradation.

### 3.1 Synthetic Routes

#### 3.1.1 Molecular Structure

The molecular structure, by altering susceptibility to water uptake and ester bond hydrolysis, plays a crucial role in the degradation rate (Table 2). Polyesters may be prepared by various synthetic strategies (e.g., condensation, ring-opening, and enzyme-catalyzed polymerizations).^96–99^ Enhanced hydrophilicity for greater water uptake may be generally imparted by reduced hydrocarbon moieties versus hydrophilic moieties.^100–103^ For instance, among common degradable aliphatic polyesters, hydrophilicity (on the basis of chemical structure) increases in the order PCL < PLA < poly­(glycolic acid) (PGA). The glass transition temperature (*T*_g_) of a polyester, also dictated by molecular structure, impacts hydrolytic degradation as amorphous chains in their rubbery state (*T* > *T*_g_) increase hydrolytic degradation rates.^104^ Molecular symmetry, by producing enhancements in crystallinity,^105^ can lead to a reduction in hydrolytic degradation rates as a crystalline lamella limits diffusion of water.^10,94^ For instance, Kumar et al. analyzed blends of amorphous poly­(d,l-lactide) (PDLLA) and semicrystalline PLLA, identifying faster degradation rates for blends with greater amounts of PDLLA compared to the neat PLLA.^106^ In some reports, crystallinity has been shown to increase during hydrolytic degradation, attributed to early hydrolytic scission of amorphous chains that permits crystallization.^82,107^ Polak-Krasna et al. showed that PLLA (*M*_n_ ≈ 98 kg/mol, ∼36% crystallinity), after 112 days in PBS, was marked by areduction in *M*_n_ (∼7.4 kg/mol) but an increase in crystallinity (∼65%). Reduced molecular weight and increased branching accelerate hydrolytic degradation by reducing crystallinity.^109^ For instance, in Roberts et al., we reported faster degradation rates of cross-linked networks prepared from *star*-PCL (“4 arm”) versus *linear*-PCL acrylated macromers, with faster degradation rates observed for those based on *star*-PCL and with reduced macromer molecular weight owing to reduced crystallinity.^110^ Wan et al. also demonstrated that reducing the molecular weight (from 900 to 300 g/mol) and hence crystallinity of 3-arm PCL led to a 2-fold increase of degradation rate in neutral conditions.^111^ Christodoulou et al.^112^ and Ponjavic et al.^113^ also analyzed different architectures of PCL using various macroinitiators and identified faster degradation rates with increased branching due to reduced crystallinity. Relative to homopolyesters, copolyesters (e.g., random, block, alternating, and graft) may also increase rates of degradation through reduced crystallinity and/or enhanced hydrophilicity.^114,115^ Among poly­(lactic acid-*co*-glycolic acid) (PLGA) copolymers of varying molar ratios of lactic acid to glycolic acid, PLGA (50:50) exhibited the fastest degradation rate due to a combination of a lack of crystallinity and substantial hydrophilicity.^116^ Yin et al. reported copolymers based on dioxanone (DO) and l-lactide (LLA) and PDO-*block*-PLLA copolymers, with increased PDO content resulting in reduced crystallinity and subsequent faster rates of degradation.^117^ In Xi et al., as trimethylene carbonated (TC) was reduced in PLLA-*co*-PTC copolymers, water uptake and degradation rates increased, owing to decreased crystallinity and increased hydrophilicity.^13^ Greater hydrophilicity alone also leads to copolymers with enhanced rates of degradation. For example, in Xia et al., PLLA-*block*-PTMC-*block*-PGA copolymers exhibited faster degradation with higher content of the more hydrophilic PGA.^118^ Yang et al. reported that copolymers of methylidene-dioxepane (MDO) and hydroxyethyl methacrylate (HEMA) [PMDO-*co*-PHEMA] degraded faster versus PCL.^119^ Seemingly minor changes in the molecular structure can also impact degradation rates. Nowalk et al. observed that *ran*-PLGA with a short-range scrambling of monomer order demonstrated faster degradation versus *alt*-PLGA with a precise alternating sequence.^120^ Overall, strategies to impart a tuned molecular structure to impact key properties (e.g., hydrophilicity, *T*_g_, and % crystallinity) can be utilized to accelerate degradation rates of polyesters.

**Table 2:** Recent Studies That Leverage Synthetic Routes to Accelerate the Degradation Rates of Polyesters

	Study	Polyesters Used	Degradation Modifier	Key Findings
**Molecular Structure**	Roberts, 2024 ^110^	PCL	Architecture, Molecular weight, Crystallinity	*Star*-PCL (4-arm) at lower MW demonstrated faster degradation rates.
	Polak-Kraśna, 2021 ^108^	PLLA	Structure	As PLLA degraded, crystallinity increased
	Christodoulou, 2022 ^112^	PCL	Architecture	As branching increased (3–5 arms), degradation rates increased
	Ponjavic, 2020 ^113^	PCL	Architecture	As branching increased (3–6 arms), degradation rates increased
	Wan, 2008 ^111^	PCL	Architecture, Molecular weight	Low MW PCL demonstrated faster degradation rates
	Kumar, 2022 ^106^	PDLLA PLLA	Crystallinity	Blends of PDLLA/PLLA degraded faster versus PLLA
	Nowalk, 2019 ^120^	PLGA	Copolymer	*ran*-PLGA demonstrated faster degradationversus *alt*-PLGA
	Xia, 2022 ^118^	PLLA-*b*-PTMC-*b*-PGA	Copolymer, Crystallinity	PLLA-*block*-PTMC-*block*-PGA exhibitedfaster degradation with increased PGA content
	Xi, 2019 ^13^	PLLA-*co*-PTC	Copolymer	PLLA-*co*-PTC copolymers degraded faster with greater PLLA content
	Yin, 2022 ^117^	PDO-*co*-PLLA	Architecture, Copolymer	PDO-*block*-PLLA copolymers degraded faster with greater PDO content
	Yang, 2023 ^119^	PMDO-*co*-PHEMA	Copolymer	PMDO-*co*-PHEMA copolymers degraded faster than PCL

### 3.2 3D Polymer Systems

#### 3.2.1 Physical and Covalent Networks

Polyesters may be physically or chemically combined to accelerate the degradation rates. The resulting reduction in crystallinity, increased hydrophilicity, and/or phase separation (i.e., immiscibility)^121–123^ play a significant role in the extent of water penetration and subsequent ester bond hydrolysis. Physical blends continue to be widely studied.^124^ Pan et al. demonstrated that PDO/PLA blends resulted in faster rates of *in vivo* degradation versus PLA.^125^ Penshne et al. reported that PBS/poly­(3-hydroxyalkanoate) (PHA) blends degraded faster versus PHA.^126^ Magazzini et al. confirmed that PGA/PCL and PGA/PLA blends degraded faster *in vitro* versus the corresponding homopolymers.^127^ In some combinations, at least one polyester is covalently cross-linked, and these may be categorized as (i) interpenetrating network (IPNs) [an interwoven polymer network comprised of two or more polymers that each form a discrete network], (ii) semi-interpenetrating networks (semi-IPN) [a polymer network containing non-cross-linked, interwoven polymer chains], and (iii) co-networks [two or more polymers collectively form a single network] ^128–130^ (Table 3). Cross-link density, by reducing both water uptake and chain mobility, is generally associated with slower degradation.^131,132^ In Pfau et al., we reported PCL/PLA semi-IPNs formed with cross-linked PCL and PLA-based thermoplastics of varying *M*_n_, crystallinity, and hydrophilicity, with accelerated degradation achieved for those exhibiting moderate phase separation.^133^ Shi et al. reported that semi-IPNs based on a PLLA-*co*-PCL network and thermoplastic PLLA, while more hydrophobic, degraded faster than the corresponding PLLA network.^134^ In Beltran et al., we demonstrated faster rates of degradation of PCL-polydimethylsiloxane (PDMS) co-networks versus PCL networks owing to phase separation effects.^135^ In summary, physical and covalent networks (e.g., blends, cross-linked networks, and semi-IPNs) of polyesters can be employed to accelerate hydrolytic degradation.

**Table 3:** Recent Studies That Leverage 3D Polymer Systems and Processing Methods to Accelerate the Degradation Rates of Polyesters

	Study	PolyestersUsed	Degradation Modifier	Key Findings
**Blends and Networks**	Pfau, 2020 ^133^	PCL/PLA	Semi-IPN,Molecular weight	Varying PLA-based thermoplastic *M*_*n,*_ hydrophilicity, and crystallinity modulated the rates of degradation
	Shi, 2024 ^134^	PLLA/PLLA-*co*-PCL	Semi-IPN	The semi-IPNs demonstrated faster degradation comparedto the PLLA networks
	Beltran, 2021 ^135^	PCL	Co-network, Architecture	PCL/PDMS co-networks degraded faster versus PCL networks
	Pan, 2021 ^125^	PDO/PLA	Blend	Blends with higher PDO content exhibited fasterdegradation
	Peshne, 2024 ^126^	PHA/PBS	Blend	Blends with higher PBS content exhibited fasterdegradation due to increased hydrophilicity
	Magazzini, 2021 ^127^	PCL/PGA PLA/PGA	Blend	Blends degraded faster versus homopolymers.
**Composites**	Nitschke, 2024 ^140^	PCL PCL/PLLA	Semi-IPN, Architecture, Composite	Semi-IPNs based on s*tar*-PCL and *star*-PLLA containing BG demonstrated faster degradation rates versus noncomposites
	Chen, 2024 ^147^	PPF	Composite	Incorporation of black phosphorus nanosheets increased degradation rate
	Guo, 2024 ^145^	PLA	Composite	Incorporation of Mg(OH)_2_ increased degradation rates
	Larijani, 2024 ^146^	PCL	Composite	Incorporation of nanoclays increased degradation rate
**Fabrication**	Khajehmohammadi, 2022 ^158^	PCL	Pore morphology	Scaffolds with star-shaped pores demonstrated faster degradation than those with gyroid-shaped pores
	Ju, 2020 ^159^	PBS	Pore morphology	Bimodal PBS scaffolds (with smaller and larger pores)degrade 10% more than unimodal scaffolds
	Dang, 2020 ^157^	PCL	Porosity	3D printed PCL scaffolds with greater intrastrut porosity demonstrated faster degradation
	Kwon, 2020 ^156^	PCL-*ran*-PLA	Fabrication	Scaffolds formed via SCPL, having greater pore heterogeneity, degraded faster than 3D printed scaffolds
**SurfaceModification**	Heydari, 2022 ^163^	PGS/PLA	Plasma treatment	Oxygen plasma-treated nanofiber films exhibited fasterdegradation
	da Silva, 2024 ^161^	PLA	Plasma etching	Argon plasma etched PLA films degraded faster due totheir increased wettability and surface roughness
	Donate, 2021 ^165^	PLA	Plasma treatment, Alkali treatment	Oxygen plasma-treated scaffolds degraded faster versus alkaline-treated scaffolds
	Ghorbani, 2019 ^167^	PCL	Coating	PDA-coated PCL scaffolds degraded faster versusuncoated scaffolds
	Azadani, 2024 ^168^	PHB	Coating	PHB-BG composite scaffolds coated with chitosan and MWCNT demonstrated faster degradation versus uncoated scaffolds
**Sterilization**	Zhao, 2019 ^170^	PLA	E-beam, EtOSS, HPGP	Sterilization with SS resulted in faster versus sterilization via EtO, E-beam, or HPGP
	Jain, 2021 ^171^	PDLAPLLA	EtO, E-beam	Versus PLLA, PDLA exhibited a greater reduction in MW following EtO and E-beam sterilization
	Kang, 2020 ^172^	PCL	E-beam	Sterilization by E-beam produced faster versus nonsterilization
	Fedorenko, 2022 ^173^	PLA	Gamma irradiation	Gamma irradiation at higher doses (75 kGy) induced faster rates of degradation.
	Chausse, 2023 ^174^	PLLAPLA-*co*-PCL	Gamma irradiation	Gamma irradiation (8 kGy) caused a significant decrease in molecular weight.
	Houk, 2021 ^175^	PCLPCL/PLLA	EtO sterilization	EtO sterilization resulted in no significant change in degradation rates

#### 3.2.2 Composites

Resorbable composites are frequently formed by combining a degradable polyester with fillers such as bioceramics^136^ (e.g., hydroxyapatite and tricalcium phosphate) and micro- and nanofillers (e.g., silicates, nanoclays, graphene, and nanotubes) to refine properties (e.g., rigidity and bioactivity).^137,138^ Compared to the corresponding neat polyester, composites may produce faster rates of degradation owing to increased water penetration stemming from greater hydrophilicity, surface area, and phase separation, as well as reduced crystallinity and *T*_g_ values.^51,136,138,139^ In Nitschke et al., we showed that composite scaffolds prepared from bioactive glass (45S5 BG) and PCL/PLLA semi-IPN networks achieved faster degradation compared to the corresponding neat scaffolds.^140^ With polyester/BG systems, the combination of acidic byproducts (due to polyester degradation) and alkalinity (due to the formation of the calcium phosphate layer) also produces a more neutral local environment *in vivo* for potential reduction in inflammation.^141–144^ Guo et al. utilized Mg­(OH)_2_ nanoparticles to increase the hydrophilicity of PLA and subsequently accelerate degradation.^145^ Larijani et al. prepared composites based on PCL and nanoclays with accelerated degradation rates.^146^ Chen et al. incorporated bioactive black phosphorus nanosheets (BPNSs) into poly­(propylene fumarate) (PPF) networks, resulting in an increase in the rate of degradation.^147^ Overall, polyester composites, formed by a combination with a vast selection of fillers and at varying concentrations, can achieve faster rates of degradation.

### 3.3 Processing Methods

#### 3.3.1 Fabrication

Fabrication methods may accelerate the polyester degradation rates through alterations to morphological features. Porous polyesters have frequently been utilized as scaffolds in tissue engineering to allow for cellular infiltration, nutrient transport, and neotissue formation.^148–150^ Porosity also accelerates polyester degradation by affording enhanced water infiltration, greater surface area, and autocatalysis.^151,152^ Key parameters of the porous structure that can affect degradation rates include pore size (including size distribution), pore volume (i.e.,% porosity), and pore morphology (e.g., interconnectivity).^153,154^ Specifically, polyesters with larger pore sizes and low porosity degrade slower compared to the corresponding polyester with smaller pores and higher porosity. Pore interconnectivity allows superior water filtration to accelerate degradation. Porous polyester scaffolds can be fabricated using techniques such as solvent-cast particulate leaching (SCPL), gas foaming, freeze-drying, thermally induced phase separation (TIPS), electrospinning, and 3D printing.^154,155^ Kwon et al. compared PCL-*ran*-PLA scaffolds fabricated using 3D printing and SCPL and showed that the latter demonstrated faster degradation, potentially due to pore size heterogeneity.^156^ Dang et al. 3D printed macroporous PCL scaffolds with varied intrastrut porosity and reported that the scaffolds with the higher strut porosity (∼35%) demonstrated faster degradation.^157^ Khajehmohammadi et al. analyzed pore geometry to optimize degradation, identifying that star-shaped pores demonstrated faster degradation than gyroid pores.^158^ Ju et al. also focused on different pore sizes and reported that bimodally sized pores (∼10–25 μm and 90–140 μm) demonstrated faster degradation compared to unimodally sized pores (∼10–20 μm) owing to an increased surface area.^159^

#### 3.3.2 Surface Modifications

Surface modifications may accelerate the degradation rates of polyesters by imparting changes to the topography and/or surface chemistry. Greater surface roughness provides more surface area to promote interaction with water and thus faster degradation rates.^160^ Surface treatments that enhance hydrophilicity aid in wettability and penetration by water.^160^ Plasma etching utilizes ions and electrons to impart both increased surface roughness and hydrophilicity.^161^ Surface roughening occurs via volatilization and with a potential reduction in molecular weight within the remaining specimens. Depending on the gaseous environment, plasma etching can enrich surfaces with various polar moieties (e.g., −COOH, −COO, −NH_2_, −OH).^162–164^ Heydari et al. demonstrated that oxygen plasma-treated poly­(glycerol sebacate) (PGS)-*co*-PLA nanofiber films exhibited accelerated degradation rates.^163^ da Silva et al. showed that argon plasma-etched PLA films resulted in accelerated degradation rates.^161^ Alkaline surface treatment with NaOH (aq.) can increase surface roughness (via erosion) and increase hydrophilicity (through enrichment with hydroxyl groups [−OH], or alternatively, carboxyl groups [−COOH] if subsequently treated with organic acids).^165,166^ However, if not closely monitored, alkaline treatment has the potential to alter the bulk properties. Donate et al. compared oxygen plasma-treated versus alkaline surface-treated 3D-printed PLA scaffolds and showed that the former demonstrated faster degradation due to the higher surface concentration of −COOH moieties as well as increased roughness.^165^ Certain coatings, by imparting greater surface hydrophilicity, have also been utilized to alter the degradation rates of polyesters. Ghorbani et al. showed that PCL scaffolds coated with polydopamine (PDA) accelerate the degradation rates.^167^ Azadani et al. showed that PHB-BG scaffolds coated with both chitosan and multiwall carbon nanotubes (MWCNTs) demonstrated slightly faster degradation rates than uncoated scaffolds.^168^ In Nitschke et al., by employing a BG/fused salt template in a SCPL method, we formed PCL/PLLA semi-IPN composite scaffolds with BG concentrated on the pore surfaces for accelerated degradation rates.^140^

#### 3.3.3 Sterilization

While sterilization is not necessarily intended to accelerate degradation, these processes can have an impact. A wide variety of sterilization techniques are available across all implanted medical devices, including steam sterilization (SS), hydrogen peroxide gas plasma (HPGP), ethylene oxide (EtO), gamma irradiation (γ-irradiation), ultraviolet (UV) radiation, and electron beam (E-beam).^169^ As expected, SS is not considered to be suitable for sterilization of polyesters. For instance, Zhao et al. showed that PLA was degraded by SS, but not by EtO, E-beam, or HPGP.^170^ Still, other sterilization techniques can cause polyester chain scission and reduced molecular weight, particularly in amorphous regions, leading to accelerated degradation upon implantation. Jain et al. reported that following E-beam and EtO sterilizations, amorphous PDLA exhibited a greater decrease in molecular weight versus semicrystalline PLLA.^171^ Kang et al. reported the E-beam sterilized PCL underwent faster *in vivo* degradation versus non-E-beam sterilized specimens.^172^ Fedorenko et al. showed that γ-irradiation of PLA, particularly at higher dosages (75 kGy), led to faster degradation.^173^ Chausse et al. showed that for PLA and PLA-*co*-PCL stents, γ-irradiation led to greater reduction in molecular weight versus EtO sterilization.^174^ In Houk et al., we showed that EtO sterilization did not have a significant effect on PCL and PCL/PLLA semi-IPN scaffolds.^175^ Overall, the impact of sterilization on polyester degradation must be assessed in advance, but sterilization methods may also be used to deliberately accelerate degradation.

## 4 Assessment of Polyester Degradation

Assessing the degradation rates as well as erosion behavior of polyesters includes numerous *in vitro* and *in vivo* methods (Figure 3). *In vitro* testing offers greater convenience, including utilization of conditions to accelerate degradation for study brevity. In accelerated conditions, however, the relative differences in degradation behavior among different polyester specimens may be minimized, and results can potentially be misconstrued. *In vitro* testing in physiological conditions (i.e., pH 7.4, 37 °C) will better capture relative differences in polyester specimen degradation, but it is still limited in its ability to precisely predict *in vivo* degradation behavior due to the complexity of the physiological environment. Herein, methods to analyze or potentially predict polyester degradation rates are highlighted.

**Figure 3:**
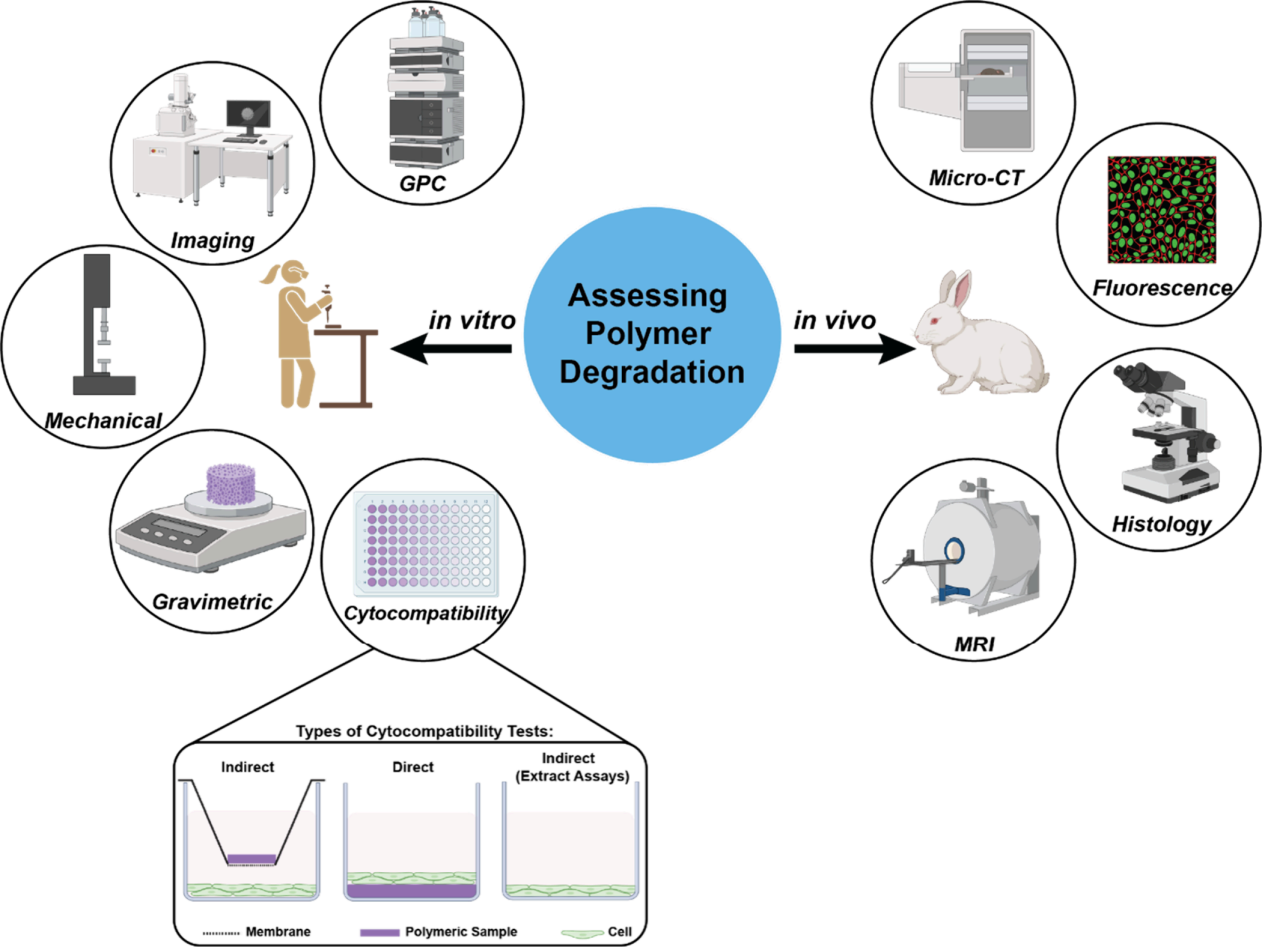
General methods for assessing polymer degradation *in vitro* and *in vivo*.

### 4.1 *In Vitro* Assessment of Degradation

ASTM F1635-24 outlines *in vitro* degradation testing parameters for surgical implants, including polyesters.^176^ To better parallel physiological conditions, tests are performed at 37 °C in buffered saline (pH 7), and the solution-to-specimen mass ratio is greater than 30:1 for adequate buffer capacity. Simulated body fluid (pH 7.4) may be used *in lieu* of buffered saline. During immersion, specimens are unloaded or may be subjected to cyclical or static loading. Since such conditions could take polyesters months or years to appreciably degrade, testing is often expedited by employing alkaline or acidic media to accelerate hydrolysis. Temperature may also be utilized to accelerate *in vitro* degradation as amorphous chains in a rubbery state (*T* > *T*_g_), and crystalline chains in a melt state (*T* > melt transition (*T*_m_)) are more permeated by water. Recent studies demonstrate the impact of elevated testing temperatures, including studies for PLA and PGA-*co*-TMC-*co*-PCL (37 °C versus 55 °C).^177^ Additionally, to induce enzyme-mediated hydrolysis, one or more enzymes may be incorporated, often at concentrations of 1–45 units/mL. *In vitro* hydrolytic degradation is most frequently assessed by monitoring temporal changes in (i) mass [eq 1] or (ii) molecular weight [eq 2].^176,178^

1 $$\mathrm{mass loss}\;\left(\%\right)=\frac{m_{i}-m_{t}}{m_{i}}\times 100$$

where *m*_i_ is the initial mass and *m*_*t*_ the mass measured gravimetrically at a designated time point.

2 $$\mathrm{degradation}\;\left(\%\right)=\frac{M_{i}-M_{t}}{M_{i}}\times 100$$

where *M*_i_ is initial molecular weight and *M*_*t*_ is the molecular weight at a designated time point.

Methods to measure molecular weight include nuclear magnetic resonance (NMR), gel permeation chromatography (GPC), and mass spectrometry.^178–180^ An example of molecular weight-based degradation studies was reported by Ribeiro et al. for poly­(glycolide-*co*-caprolactone) (PGCL) and poly­(glycolide-*co*-lactide) (PGL).^181^ After 21 days in neutral media, PGCL demonstrated a 3% degradation (*M*_n,initial_ ≈ 47 kDa to *M*_n,final_ ≈ 46 kDa) while PGL showed a 31% degradation (*M*_n,initial_ ≈ 56 kDa to *M*_n,final_ ≈ 39 kDa).

As mentioned above, numerous limitations of *in vitro* degradation studies exist. Among reported studies, there is tremendous variability in utilized media (e.g., molar concentration of basic or acidic media), media volume, and specimen dimensions. Collectively, this makes it difficult to broadly compare the polyester degradation data among various reports. Additionally, most studies assess mass loss or molecular weight reduction rather than both. However, each provides distinct and essential information. Mass loss gives insight into when notable weight loss occurs, which could greatly impact the mechanical integrity. Given the molecular weight limit for oligomers able to be excreted from the body,^182–184^ monitoring of molecular weight is also useful. It is also essential to assess other properties that accompany polyester degradation, including the accompanied change in mechanical and/or rheological properties.^176,185^ Erosion should be assessed to reveal the nature of mass loss (e.g., cracks, pits, and/or surface roughening), using methods such as scanning electron microscopy (SEM).^52,178,186,187^ Transmission electron microscopy (TEM) can provide additional information on the changes to the internal microstructure, such as the crystallinity and phase separation within the polyester.^187^ More recently, computational approaches have recently been utilized to create predictive models of the degradation of polyesters and other polymers, including machine learning (ML).^188–193^ A major barrier is the lack of available large data sets for training and testing of ML models.^194^ Overall, numerous factors should be considered to effectively evaluate the hydrolytic degradation of polyesters with *in vitro* experimental and computational approaches.

In addition to these *in vitro* assessments of degradation, cytotoxicity tests are often conducted. ISO 10993-5:2009 describes methods to assess cytotoxicity using cultured cells in terms of cellular adhesion, viability, and proliferation.^195^ Cultured cells may be exposed to the material via direct or indirect tests. In the case of degradable polyesters, indirect contact would permit exposure to degradation byproducts and so is a useful method. Cellular response to materials may be evaluated qualitatively via microscopy (e.g., morphology observed with a light microscope) or quantitatively via viability assays (e.g., LIVE/DEAD and MTT assay). It is important to note that a material’s cytocompatibility, while an aspect of biocompatibility, is limited to a lack of harmful effects toward cells.^196,197^ Biocompatibility further encompasses a lack of a negative host response with respect to tissues, organs, and the immune system and so is assessed via *in vivo* analyses.

### 4.2 *In Vivo* Assessment of Degradation

Owing to contributions of a complex physiological environment, *in vivo* degradation of polyesters is often observed to be relatively faster than *in vitro* (buffered saline, 37 °C).^198^ Per ISO 10993-6:2016, initial *in vivo* evaluation of biodegradable polymers is frequently performed with a small animal model (e.g., mouse, rat, and rabbit).^199^ Larger animal models (e.g., pig, sheep, and goats) may be justified for long-term studies and when a larger or whole device is evaluated. These analyses assess local tissue effects following subcutaneous implantation, with comparisons made to a control material considered clinically acceptable and with established biocompatibility characteristics.^200^ Assessment of degradation is frequently performed on explanted specimens. Histological evaluation provides information on the response of the local tissues and potentially may also capture material degradation in terms of erosion.^201^ For example, Nettleton et al. utilized histology staining (hematoxylin and eosin (H&E) and Goldner’s Trichrome) to verify tissue ingrowth and degradation of bone scaffolds on a scale of 0–4 (no degradation to abundant degradation, respectively); after 12 weeks *in vivo*, PPF scaffolds showed degradation (scaled ∼2.5–3.0).^202^ Dias et al. also used histology (H&E) to analyze electrospun PCL meshes *in vivo* and identified voids indicative of degradation.^203^ However, histology remains limited in providing quantitative metrics of degradation. Therefore, to assess polyester degradation, noninvasive methods to quantitatively and temporally analyze *in vivo* degradation (as well as *in vitro* degradation), such as magnetic resonance imaging (MRI), fluorescence imaging, and microcomputed tomography (micro-CT), are used.^178,200,204,205^ MRI can provide high temporal and spatial resolution, with contrast agents enabling an opportunity to decipher the polymer implant from surrounding tissue.^206^ For instance, Yang et al. utilized MRI to monitor *in vivo* degradation of Fe_3_O_4_-nanoparticle-loaded PLA-gelatin electrospun scaffolds in a rat model.^207^ Fluorescent imaging, particularly using near-infrared (NIR) imaging to mitigate tissue absorbance, can be used to monitor polymer degradation by incorporation of fluorescent probes.^208,209^ For instance, Kim et al. utilized NIR imaging to evaluate the *in vivo* degradation of NIR-conjugated PCL-*ran*-PLLA-*ran*-PGA scaffolds.^210^ Micro-CT produces a 3D image by reconstructing a series of 2D X-ray images obtained at different angles and may be performed on polymer implants that contain radiopacifiers.^211^ For instance, Perez et al. reported the use of micro-CT to monitor the degradation of iodixanol/PCL fibrous scaffolds in a rat model.^212^ In combination, multimodal approaches represent opportunities for improved analysis.^213,214^ For instance, Chen et al. combined ultrasound, fluorescence, and MRI to study the *in vivo* degradation of PLGA-*block*-PEG-*block*-PLGA.^215^ While these strategies provide highly informative insights into polyester *in vivo* degradation, the associated use of animal models and significant costs demand that *in vitro* analyses be leveraged to optimize success.

## 5 Polyesters in Biomedical Applications

The use of polyesters within biomedical engineering extends across many applications, including regenerative engineering, drug delivery, and medical devices (Figure 4, Table 4). As noted previously, accelerating the rate of polyester degradation is essential to many of these applications. Some contemporary examples of polyesters utilized in these biomedical applications are highlighted herein.

**Figure 4:**
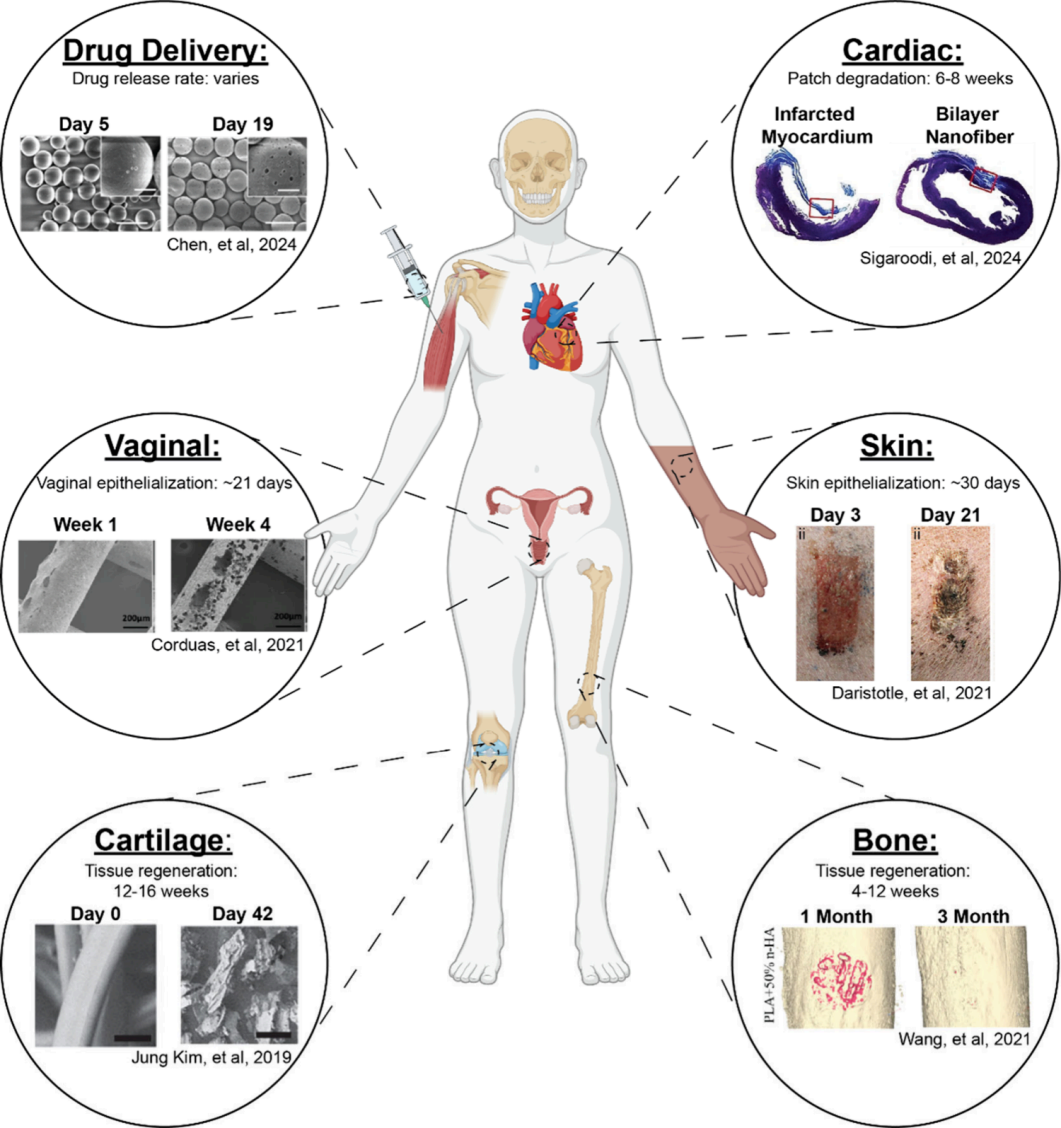
Utility of biodegradable polyesters in various biomedical applications.

**Table 4:** Recent Studies of Polyesters with Accelerated Degradation Rates for Targeted Biomedical Applications

Application	Study	Compositions	Degradation Modifier	Degradation Testing Method	Fabrication Method	Key Findings
**Bone Tissue**	Daskalakis, 2023 ^223^	PCLPCL/HAPCL/TCPPCL/BG	Composite, Pore size	*In vitro* (Mass loss – *aq*. NaOH)	3D printing (AM)	PCL/BG scaffolds demonstrated the fastest rate of degradation. Scaffolds with larger pore sizes degraded faster.
	Wang, 2021 ^224^	PLAPLA/nano-HA	Composite	*In vivo* (micro-CT)	3D printing (FDM)	Due to faster rates of degradation (and bioactivity), PLA/nano-HA scaffolds demonstrated greater neo-tissue ingrowth versus PLLA scaffolds.
	Zhang, 2023 ^234^	PCL-*g*-PLGAd	Copolymer, Co-network, Surface coating, Composite	*In vivo* (Histology)	SCPL	PLGA–PCL/PPDLDA coated in PDA and HA allowed for *in vivo* degradation with 68% mass remaining after 3 months
	Xu, 2024 ^235^	PPFPPF-*co*-PCL	Copolymer, Composite	*In vivo* (Histology)	Injectable	PPF-*co*-PCL scaffolds degraded faster versus PFF scaffolds, resulting in greater neotissue.
	Ghorbani, 2021 ^236^	PBP	Composite, Plasma treatment	*In vitro* (Mass loss – PBS)	Freeze cast	Oxygen plasma-treated PBP scaffolds degraded nearly completely.
**Cartilage Tissue**	Baranowski, 2022 ^243^	PLLA-*co*-PCL	Copolymer, Fabrication	*In vivo* (Histology, GPC)	Porogen leaching	Explanted scaffolds exhibited significant reduction in *M*_w_ at 8 and 16 weeks
	Savin, 2024 ^244^	PEU	Blend, Coating	*In vitro* (Mass loss – PBS*)*	Electrospinning	The collagen-coated PEU scaffold exhibited 40% mass loss in 2 months versus a PCL scaffold with 0% mass loss in 1 year
	Jung Kim, 2019 ^245^	PLGA	Copolymer	*In vitro* (Mass loss – PBS)	Wet spinning	PLGA fibers loaded with BMPs degraded in 42 days, and promoted cartilage regeneration in a rabbit model at 6 weeks.
**Women’s Health**	Leprince, 2019 ^248^	PDLA-*b*-PEO-*b*-PDLA	Copolymer	*In vivo* (Visual inspection)	–	Films degraded in 3–10 days with the fastest degradation occurring when PEO content was the highest.
	Wu, 2023 ^255^	PCL/SF	Blend	*In vitro* (Mass loss - enzymatic)	3D printing (EHDP)	Incorporating silk fibroin accelerated the degradation rate of PCL meshes
	Corduas, 2021 ^252^	PCLPCL/0.5LFX	Composite	*In vitro* (Mass loss – *aq*. NaOH)	3D printing	The 3D-printed vaginal meshes with Levofloxacin, a hydrophilic drug, demonstrated accelerated degradation
	Magalhaes, 2020 ^249^	PLGA-coated PGA	Coating	*In vivo* (Histology)	–	Scaffolds underwent complete degradation after 3 months.
	Subramanian, 2020 ^258^	PCL/PEG	Semi-IPN,Composite	*In vitro (Mass loss - SUF)*	Solvent cast	PCL compositions with more SMA showed longer degradation rates
**Drug Delivery**	Behnke, 2024 ^263^	PLGAPValGPEtOx-*b*-PValG	Copolymers	*In vitro* (Mass loss - enzymatic)	HT formulation	PEtOx-*b*-PValG nanoparticles demonstrated accelerated degradation due to increased hydrophilicity and surface area
	Liao, 2024 ^264^	POCPOL	Copolymers, Molecular weight	*In vitro* (Mass loss – PBS)	Injectable	POL exhibited faster degradation due to increased hydrophilicity.
	Chen, 2024 ^262^	PLGA-*b*-PEG/PLGA	Copolymer, Molecular weight, Pore morphology	*In vitro* (GPC)	Microfluidic chip	Microspheres with greater PLGA–PEG content and lower *M*_n_ led to faster degradation
**Cardiac Patches, and Cardiovascular Tissue**	Huang, 2024 ^268^	PMECL	Copolymer, Surface coating	*In vitro* (Mass loss - PBS/enzymatic)	3D printing (FDM)	PPy-coated PMECL patches prepared with the lowest concentration of pyrrole exhibited the fastest degradation
	Atya, 2021 ^269^	PGSPGA/CA	Composite	*In vitro* (Mass loss – Ringer solution)	–	PGA/carbon aerogel (CA) composites exhibited faster degradation versus analogous PGA without CA
	Gürbüz, 2024 ^273^	PCLPCL/PGSPCL/PGS/PSF	Blend	*In vitro* (Mass loss – PBS)	3D printing (STL)	Blends exhibited faster degradation versus PCL-only controls.
	Sigaroodi, 2024 ^270^	PCLPCL/MWCNTPCL/PXSPCL/PXS/MWCNT	Composite, Blend	*In vitro* (Mass loss – PBS/DMEM)	Electrospinning	PCL/PXS/MWCNT mats exhibited the fastest rate of degradation.
**Medical Devices**	Daristotle, 2021 ^287^	PLA-*co*-PCL	Copolymer, Molecular weight	*In vitro* (GPC)	Solution blow spinning	Blends (50:50) of high and low *M*_n_ copolymers accelerated degradation.
	Miao, 2021 ^277^	PGAPLGA	Copolymer, Postfabrication annealing	*In vitro* (Mass loss - PBS)	3D printing (extrusion)	Lower annealing temperatures accelerated degradation by reducing crystallinity
	Szabelski, 2024 ^276^	PGAGlyconatePLGAPDO	Copolymers	N/A	N/A	SafilQuick+ (made of PGA) exhibited the fastest resorption time (and greatest loss of tensile strength) while MonoPlus (made PDO) exhibited the longest resorption time
	Liu, 2021 ^278^	PLGAPPDOPLGA/PPDO	CopolymerBlend network	*In vitro* (Mass loss - PBS)	Solvent casting	PPDO/PLGA blend (70:30 wt %) demonstrated the fastest degradation.
	He, 2024 ^288^	PEG–PLA	Molecular weightCopolymer	*In vitro (Mass loss - PBS)*	Solvent casting	PEG–PLA (4k and 1k, respectively) exhibited the fastest degradation.
	Zhao, 2022 ^282^	PLA-*co*-PCL	Copolymer	*In vitro(Mass loss – PBS)*	3D printing(extrusion)	PLA-*co*-PCL (95/5) exhibited the fastest degradation

### 5.1 Regenerative Engineering of Bone Tissue

Bone defects can result from injury, tumor resection, and congenital anomalies.^216^ While autografts are commonly used to repair bone defects, they are limited by donor site morbidity and premature graft resorption.^217,218^ Regeneration with a porous polymeric scaffold is a promising alternative, but this requires a scaffold whose degradation rate parallels neotissue formation and vascularization (∼4–12 weeks) for optimal healing.^219–222^ Thus, efforts have been made to accelerate the degradation rate of polyester-based bone scaffolds using the strategies noted above (Figure 2). Daskalakis et al. prepared PCL composite scaffolds with different bioceramics (e.g., hydroxyapatite (HA), BG, or tricalcium phosphate (TCP)) and of varying pore sizes using additive manufacturing (AM).^223^ All PCL composite scaffolds degraded faster than the PCL-only scaffolds, with the PCL/BG composite scaffold demonstrating particularly accelerated degradation and mechanical robustness. Wang et al. reported that PLA/nano-HA scaffolds (formed via fused deposition modeling (FDM)), owing to accelerated degradation and increased bioactivity versus analogous PLA scaffolds, demonstrated increased markers of osteogenesis as well a full tissue infiltration (3 months; rabbit model). Several approaches have been reported to develop polyester scaffolds capable of filling irregular defects and/or for minimally invasive delivery, including shape memory polymer (SMP) scaffolds^225–227^ and injectable scaffolds.^228,229^ We have reported PCL-based SMP scaffolds that are capable of “self-fitting” into irregular bone defects following exposure to warm saline, resulting in better scaffold-to-tissue contact to promote healing.^230–233^ Zhang et al. reported SMP scaffolds for aveolor defects based on PCL-*graft*-poly­(l-glutamic acid) (PCL-*graft*-PLGAd) copolymerized with an acryloyl chloride grafted poly­(ω-pentadecalactone) (PPDLDA).^234^ In a rabbit model, the implanted PCL-*graft*-PLGAd/PPDLDA scaffold, which was also coated with PDA/silver/HA for antibacterial properties, demonstrated ∼30% weight loss in 3 months and neotissue formation. Xu et al. prepared injectable PPF and PPF-*co*-PCL scaffolds that contained PLGA microspheres loaded with vascular epithelial growth factor (VEGF) for spinal fusion, and PPF-*co*-PCL scaffolds exhibited enhanced degradation and neotissue formation at 12 weeks in rabbit models.^235^ Ghorbani et al. reported that oxygen plasma treated PCL/BG (PBP) scaffolds exhibited near complete *in vitro* degradation at 5 weeks (PBS, 37 °C).^236^

### 5.2 Regenerative Engineering of Cartilage Tissue

Loss of articular cartilage,^237,238^ due to injury or disease, requires intervention owing to its avascular nature and low capacity to heal.^239^ Cartilage regeneration using polymeric scaffolds is a promising alternative to autografting and focal resurfacing devices toward avoiding total joint replacement.^237,240^ Studies point to a period of ∼12–16 weeks for cartilage healing with acellular hydrogels,^241,242^ so recent efforts have sought to align the rate of polyester scaffold degradation. Baranowski et al. reported that PLLA-*co*-PCL scaffolds, prepared with a polyvinylpyrrolidone (PVP) porogen, exhibited two key stages of degradation at 8 and 16 weeks leading to regeneration of articular cartilage in rabbits.^243^ Savin et al. created a collagen-coated, polyester-urethane (PEU) scaffold from a PLGA and PCL-lysine diisocyanate (PCL-LDI) for use in meniscal repair and compared them to the commercially available PCL-based scaffold (Actifit).^244^ *In vitro* (PBS, 37 °C), the PEU scaffold exhibited ∼40% mass loss in 2 months while the PCL-based scaffold exhibited no mass loss in one year. Kim *et al*. utilized fibrous PLGA scaffolds loaded with bone morphogenic proteins (BMPs) and synovium-resident mesenchymal stem cells (synMSCs) that degraded within 42 days (PBS, 37 °C) and promoted cartilage regeneration within 6 weeks in rabbit models.^245^

### 5.3 Women’s Health

While historically understudied, research in women’s health has begun to attract greater attention in biomedical research.^246,247^ Biodegradable polyesters have emerged as an important class of materials to address these unmet needs. This includes the treatment of various gynecologic, reproductive, and other issues. Leprince et al. created films from copolymers based on PDLA and poly­(ethylene oxide) (PEO) [PDLA-*block*-PEO-*block*-PDLA] to prevent trauma-induced intrauterine adhesions to the endometrium by promoting sufficient re-epithelialization in ∼21 days.^248^ *In vivo* degradation rates (rat model) were between 3 and 12 days, depending on monomer ratios. Magalhaes et al. studied PLGA-coated PGA scaffolds, seeded with autologous cells, to regenerate uterine tissue necessary to support live births.^249^ In a rabbit model, scaffolds exhibited complete degradation at 3 months with the development of an epithelial lining and, at 6 months, led to vascularized uterine tissue. Pelvic floor disorders (e.g., pelvic organ prolapse (POP) and urinary incontinence) have been historically treated with nonabsorbable, transvaginal polypropylene (PP) meshes.^250,251^ However, recent FDA safety warnings have prompted the development of meshes that are biodegradable and better parallel the stiffness of vaginal tissue.^252–254^ Corduas et al. produced a drug-loaded-PCL-based vaginal mesh that degraded faster than “drug-free” meshes owing to the acidic nature of the drug that accelerated ester bond hydrolysis.^252^ Wu et al. created a degradable vaginal mesh based on blends of PCL and silk fibroin (SF), with faster degradation rates, greater collagen production, and superior tensile strength versus analogous PCL meshes.^255,256^ Hicks et al. reported a self-expanding vaginal stent based on a PCL SMP for the potential treatment of vaginal stenosis following pelvic radiation and vaginal reconstruction, and the aforementioned strategies to accelerate degradation may be of future use.^257^ In the area of contraceptives, Subramanian et al. developed a PCL/poly­(ethylene glycol) (PEG)-based, nonhormonal, degradable intrauterine device (IUD) loaded with styrene maleic anhydride (SMA), a sperm-killing hydrogel that was resorbed after 150 days *in vivo* (rat model).^258^

### 5.4 Drug Delivery

Degradable polyesters are frequently used to create drug delivery systems (DDS) to regulate the rate of drug release for improved safety and efficacy.^138,259^ Passive drug release profiles are intrinsically linked to the hydrolytic degradation rates of the polyester, as well as factors such as size and geometry of the device.^138,260^ Drug release follows a triphasic profile controlled by diffusion, erosion, or a combination of both and is characterized by three stages: (i) initial burst release of drugs, (ii) lag phase during erosion, and (iii) secondary release based on bulk erosion.^261^ In some scenarios, a relatively fast rate of drug release is desirable, necessitating a faster rate of polyester degradation. Chen et al. developed monodispersed risperidone-loaded PLGA-*block*-PEG/PLGA microspheres with tailored surface morphologies (i.e., porous structures).^262^ Versus PLGA microspheres, those containing PLGA–PEG of increased levels displayed increased porosity and water permeability, leading to faster degradation. Behnke et al. incorporated hydrophilic poly­(2-ethyl-2-oxazoline) (PEtOx) with poly­(l-valine-glycolic acid) (PValG) to form PEtOX-*b*-PVALG nanoparticles whose degradation rates were accelerated versus PLGA and PValG nanoparticles, resulting in faster release of anti-inflammatory drugs.^263^ Liao et al. reported fast-degrading poly­(ortho ester)-oligocaprolactone (POC) and poly­(ortho ester)-oligolactide (POL) injectable systems for rheumatoid arthritis, with those formed with low molecular weight POL exhibiting the most rapid *in vivo* degradation rates (∼21 days; rat model).^264^

### 5.5 Cardiac Patches and Regenerative Engineering of Cardiac Tissue

The poor regenerative potential of cardiac tissues has limited the treatment of various heart diseases. Cardiac patches based on degradable polyesters have been evaluated to restore damaged myocardium by providing temporary mechanical support.^265^ Ideally, such patches should degrade within 6–8 weeks to coincide with myocardial tissue repair.^266,267^ Huang et al. developed cardiac patches from poly­(methylcaprolactone)-*block*-PCL-PEG-PCL-*block*-poly­(methyl-caprolactone) (PMECL) and a polypyrrole (PPy) conductive coating based on different concentrations of aqueous solutions of pyrole (0.01, 0.05, and 0.1 mol/mL).^268^ Patches coated with the lowest coating concentration degraded the fastest *in vitro*, which was attributed to the reduction of the water barrier imparted by the coating. Atya et al. reported cardiac patches based on PGS and conductive carbon aerogel (CA) that degraded faster *in vitro* versus PGS-only patches and also exhibited superior myoblast adhesion and proliferation.^269^ Sigaroodi et al. created a PCL/poly­(xylitol sebacate)-PCL (PXS-PCL) bilayered nanofibrous mat loaded with conductive multiwall carbon nanotubes (MWCNT) that exhibited faster degradation *in vivo* (rat model) versus PCL controls.^270^ Heart valve tissue engineering may also utilize degradable polyester scaffolds.^271,272^ Gürbüz et al. reported PCL/PGS and PCL/PGS/polysulfone (PSF) scaffolds for increased mechanical durability that degraded faster and improved regeneration versus PCL-only scaffolds.^273^

### 5.6 Medical Devices

Degradable polyesters have been utilized for use in various resorbable medical devices (e.g., sutures, stents, and wound dressing), and degradation rates are critical to success. Biodegradable polyester sutures have been extensively used in different clinical applications (e.g., cardiac, ophthalmic, and soft tissue), and the rate of degradation has been of particular focus.^274,275^ Szabelski et al. evaluated the degradation-induced loss of tensile strength of five commercially available, degradable sutures prepared from different polyesters: SafilQuick+ (PGA), Monosyn, and Monosyn Quick – (Glyoconate: PGA-*co*-PCL-*co*-PTC), Novosyn (PLGA), and MonoPlus (PDO).^276^ SafilQuick+ demonstrates the fastest resorption time (∼42 days per manufacturer’s specification) and produced a significant loss of tensile strength within 12 days (Ringer’s solution). On the other hand, MonoPlus resorbs the slowest (∼210 days per the manufacturer’s specification) and did not demonstrate a loss of tensile strength. Fiber annealing is an important aspect of suture formation. Miao et al. demonstrated that lower annealing temperatures for PGA and PLGA provided higher degradation rates due to reduced crystallinity.^277^ For faster-resorbing sutures, Liu et al. proposed blends of poly­(para-dioxanone) (PPDO) and PLGA (70/30 wt %).^278^ Resorbable cardiovascular stents are of interest to reduce neointimal hyperplasia and restenosis associated with permanent, high modulus metals.^279,280^ The degradation rate should ideally parallel vascular tissue remodeling to afford vessel patency following resorption.^281^ Commercial biodegradable stents are frequently prepared from PLLA, PDLLA, and desaminotyrosine polycarbonate (PTD-PC) scaffolds. Recent efforts have focused on designs that afford the requisite mechanical properties (e.g., radial strength) as well as degradation profiles. For instance, Liu et al. reported that 3D printed stents prepared from PLLA-*co*-PCL (95:5) exhibited similar mechanical properties but faster degradation rates versus those prepared from PLLA.^282^ Wound healing devices (i.e., bandages and wound dressings) can also be fabricated from biodegradable polyesters.^283,284^ While healing rates tend to depend on the severity of the wound, skin can re-epithelialize in ∼21–30 days.^285,286^ Daristotle et al. fabricated biodegradable bandages based on 50:50 blends of high and low molecular weight PLA-*co*-PCL that promoted healing of a partial thickness wound (porcine model).^287^ The use of degradable polyesters has been explored for a myriad of other medical devices. For instance, He et al. proposed a resorbable esophageal stent to expand upon implantation and release anticancer therapeutics using a shape-memory PEG–PLA multiblock copolymer, wherein tuning the molecular weight of PEG and PLLA blocks (4 and 1 kDa, respectively) resulted in the fastest degradation in simulated intestinal fluid.^288^

## 6 Conclusions

Approaches to developing polyester systems with accelerated rates of biodegradation hold vast potential to advance their success in numerous biomedical applications. Representing a potential shift from conventional polyesters, recent reports noted herein highlight the focused development of faster-degrading polyester systems for regenerative engineering, drug delivery, women’s health, and other medical devices. Several general methods to impart accelerated degradation to these and other polyester systems have emerged. For instance, synthetic routes have been used to tailor molecular structure to impact key properties known to control ester bond hydrolysis (e.g., hydrophilicity, *T*_g_, and % crystallinity). A network or composite design may also be effective. Finally, processing methods that impart morphological features or modify surfaces, including sterilization techniques, can accelerate degradation rates. A robust assessment of the rate of polyester degradation and erosion, both *in vitro* and *in vivo*, is necessary. Key challenges remain in the development and clinical translation of more quickly degrading polyesters for biomedical applications. A central challenge is how the polyester degradation is characterized. Cross-comparing degradation studies is limited by the variability reported among *in vitro* testing procedures (e.g., specimen size, temperature, accelerated conditions, duration, sterilization, and characterization methods). Variability likewise exists among *in vivo* testing protocols. With improved standardization across the field, polyesters with higher degradation rates can be better targeted and evaluated for a desired application. Machine learning and techniques that afford longitudinal *in vivo* analysis could also be transformative, as well as multimodal *in vivo* methods. Resolving these issues will be critical to the efficacy and safety of the final device or therapeutic. Overall, the future of biodegradable polyesters holds great potential for numerous biomedical applications. Consistent and improved methods to assess and predict degradation rates are paramount to realizing this potential.
